# Echo chambers and opinion dynamics explain the occurrence of vaccination hesitancy

**DOI:** 10.1098/rsos.220367

**Published:** 2022-10-12

**Authors:** Johannes Müller, Aurélien Tellier, Michael Kurschilgen

**Affiliations:** ^1^ Centre for Mathematical Sciences, Technische Universität München, Munchen, Germany; ^2^ Section of Population Genetics, Technische Universität München, Munchen, Germany; ^3^ Institute for Computational Biology, Helmholtz Center Munich, Neuherberg, Germany; ^4^ Department of Economics, UniDistance Suisse / FernUni Schweiz, Brig, Switzerland; ^5^ Max Planck Institute for Research on Collective Goods, Bonn, Germany; ^6^ Stanford Graduate School of Business, Stanford, CA, USA

**Keywords:** vaccination hesitancy, opinion dynamics, reinforcement model, data analysis

## Abstract

Vaccination hesitancy is a major obstacle to achieving and maintaining herd immunity. Therefore, public health authorities need to understand the dynamics of an anti-vaccine opinion in the population. We introduce a spatially structured mathematical model of opinion dynamics with reinforcement. The model allows as an emergent property for the occurrence of echo chambers, i.e. opinion bubbles in which information that is incompatible with one’s entrenched worldview, is probably disregarded. We scale the model both to a deterministic limit and to a weak-effects limit, and obtain bifurcations, phase transitions and the invariant measure. Fitting the model to measles and meningococci vaccination coverage across Germany, reveals that the emergence of echo chambers dynamics explains the occurrence and persistence of the anti-vaccination opinion in allowing anti-vaxxers to isolate and to ignore pro-vaccination facts. We predict and compare the effectiveness of different policies aimed at influencing opinion dynamics in order to increase vaccination uptake. According to our model, measures aiming at reducing the salience of partisan anti-vaccine information sources would have the largest effect on enhancing vaccination uptake. By contrast, measures aiming at reducing the reinforcement of vaccination deniers are predicted to have the smallest impact.

## 1. Introduction

For highly transmissible infectious diseases like measles, meningitis and COVID-19, vaccine hesitancy represents a major threat to public health [1–4], preventing countries from reaching herd immunity. The reasons for people deciding not to get vaccinated (or not to vaccinate their children) range from underestimating the risk of contracting the disease and overemphasizing the vaccine’s potential side-effects to general distrust in medical professionals and public health officials [5–9]. In order to develop effective policy tools to tackle vaccination hesitancy, it is therefore of prime importance for public health authorities to understand the dynamics of an anti-vaccine opinion in the population. In this paper, we introduce a mathematical model of opinion dynamics with reinforcement and spatial structure, which allows for the emergence of echo chambers, i.e. opinion bubbles in which information that is incompatible with one’s entrenched worldview, is probably disregarded. We conceive reinforcement as a form of social influence in which individuals’ opinions are sticky because the influence of like-minded individuals (who confirm a person’s opinion) is stronger than the influence of opposite-minded individuals (who trigger doubts, and might lead to a change of mind). Reinforcement captures both homophily (people tend to interact with persons who are similar to themselves) as well as behavioural biases in people’s processing of information (people tend to discount or even dismiss information that contradicts their entrenched worldview). We not only analyse the model, but also test the model empirically.

The mathematical modelling of vaccine hesitancy has a long tradition. Similar to the incidence of an infection, vaccination decisions are based on social behaviour (see for example the review articles [10,11]). However, there is a fundamental difference: whereas getting infected is a function of exposure and (bad) luck, refusing or accepting vaccination is, provided sufficient supply of vaccines, a deliberate decision. Since the seminal work of Fine & Clarkson [12], a game-theoretical approach prevails. The individual aims to minimize their personal risk whereas public health authorities wish to improve the overall health status of the population (e.g. minimizing the disease burden [13]). When these two objectives stand in conflict (e.g. if side-effects of a vaccine are known or presumed), individuals may refuse vaccinations, which—from a public health perspective—is undesirable [12,14–17].

A more recent strand of the literature on vaccine hesitancy bypasses utilitarian considerations and focuses directly on opinion dynamics [18–20]. Rather than modelling individual decisions as a result of utility maximization, the individual is seen as being influenced by the social environment, that is the behaviour of one’s fellow citizens, news coverage and public health recommendations. This framework has been taken up by the social learning theory for vaccine hesitancy [21–23]. These studies either focus on data analysis, or use generic models to address social learning; however, when addressing the link between opinion dynamics and vaccination hesitancy [18,24–26], most studies do not test the models by data analysis. Notable exceptions are [20], which shows that spatial clusters of local outbreaks can be explained by clusters of low vaccination coverage, presumably caused by local opinion dynamics, and [27], which compares empirical and simulated clustering of pro/anti-vaccination opinions in a network. However, extant models lack an underlying mechanism for opinion dynamics and its change over time.

More generally, the presence and dynamics of vaccine hesitancy in a population is a specific case of modelling opinion dynamics over time. The basis of most opinion models, the voter model [28], has the disadvantage that in a finite, well-mixed population, only one opinion remains in the long run, which obviously is not in line with the empirical reality. A remedy is the introduction of zealots or stubborn individuals which decreases, while not fully preventing, the occurrence of opinion loss [29–33]. Yet, the zealot model does not incorporate mechanisms that lead to the formation of echo chambers. Instead, nonlinear opinion models may account for such effects (see the Sznajd model [34,35], the Ising model [36], the Axrelrod model of cultural diffusion [37], or the name game [38,39] to name but a few). A different class of models do not assume two opinions (e.g. pro or contra vaccination), but the quantitative opinion of a person is represented by a real number (see review article [40]). In the bounded confidence model [41], only individuals with an opinion close enough can interact. The nonlinearity introduced in this way also is able to create echo chambers, and—as we will see—the present approach has a similar spirit.

While extant nonlinear models are rather phenomenological and not behaviourally tested by empirical studies (with few exceptions, such as [42]), the reinforcement model introduced here aims to implement behavioural mechanisms which have been extensively documented in psychology, economics and political science. In our model, zealots represent people’s exposure to stubborn individuals, i.e. characterized as exhibiting partisan (pro-vaccine or anti-vaccine) information. Controlled behavioural experiments show that distorted information critically affects people’s decision making such as the willingness to cooperate [43]. Recent field evidence finds that during the current COVID-19 crisis, higher exposure to Fox News reduces people’s propensity to follow stay-at-home orders [44].

In addition, our model accounts for the fact that individuals are disproportionately exposed to ideas similar to their own. In other words, their ideas are being reinforced. Behavioural research has documented two main sources of reinforcement. On the one hand, there are technical filters of information. Both traditional media outlets and social networks have incentives to align their stories to the (presumed) opinions of their readers and users [45–47]. However, even in social networks individuals are usually exposed to a large range of information [48,49]. In addition to technical filters, information is being filtered—often subconsciously—by the individuals themselves [50–52]. People are more likely to dismiss information that is in conflict with one’s entrenched worldview [53,54], as well as information from sources that are perceived as outgroup [55,56]. In particular, political partisanship has been found to have a sizable effect on people’s acceptance of government vaccination recommendations. Democrats were much more likely than Republicans to believe in the safety of the swine flu (H1N1) vaccine, introduced in 2009 by the Obama administration. By contrast, Republicans were much more likely than Democrats to believe in the safety of the smallpox vaccine, introduced in 2003 by the Bush administration [57]. In the current COVID-19 crisis, Democrats are more likely to believe in the perils of contracting the virus, and in the effectiveness of social distancing [58].

Vaccination data exhibit an inherent spatial structure, evidenced, for example, by the correlation between local outbreaks and vaccination coverage [20]. Indeed, the vaccination rates of the two diseases studied here, measles and meningococci, show strong spatial patterns across Germany [59]. Districts that are geographically closer exhibit more similar vaccination coverage for both diseases than districts that are geographically more distant. Furthermore, these correlations could not be appropriately explained by social co-factors like unemployment, or income [59]. To account for this inherent spatial structure, we develop and analyse a spatially structured variant of our model, based on the simple assumption that communication between individuals—and thus transmission of vaccination opinions—not only occurs *within* a local geographical area but also *between* neighbouring areas.

In the Methods section, we summarize for non-mathematical readers the main principle of our reinforcement model; the technical details can be found in the electronic supplementary material. We then provide a more detailed description of the model and of the vaccination coverage data used. In the Results section, we first analyse the model behaviour analytically by scaling to a deterministic limit and discuss the bifurcation structure in a relatively simple two-patch system. Subsequently, we scale the model to a weak effects limit, which yields a Fokker–Planck equation, and obtain the invariant measure. In the second part of the Results section, we use this invariant measure to analyse vaccination data for measles and meningococci in Germany. We show that the model is appropriate, and use the estimations as a basis to predict the effectiveness of various policies aimed at increasing vaccination uptake.

## 2. Methods

### 2.1. Mathematical model in a nutshell

To study the opinion dynamics of vaccine hesitancy, we develop a spatial, i.e. graph-based, opinion model with reinforcement. This model allows as an emergent property for the occurrence of echo chambers, in which persons with similar opinions cluster and avoid effective interaction with individuals of the opposite opinion, the strength of which depends on the degree of contact between individuals and the probability to change one’s mind upon contact.

The graph structure is used to model space. There is an important difference to many graph-based opinion models that have social networks in mind. In those models, a node represents a single person. In our case, a node represents a district, with within-district and between-district communication. This set-up allows us to go to a (deterministic or stochastic) continuum limit within each node.

Reinforcement allows for the emergence of echo chambers, in that persons with similar opinions cluster and avoid to effectively interact with individuals of the opposite opinion. These are environments in which individuals’ (pro-vaccine or anti-vaccine) opinions get reinforced through biased interaction with peers or exposure to sources with similar tendencies and attitudes as themselves [60]. Our reinforcement model builds on the classic voter model [28]. Each individual is assumed to be born with a certain opinion (pro-vaccine or anti-vaccine). In order to change their opinion, individuals need to be exposed to the opposite opinion. In this case, an individual has a certain probability to change their current opinion.

We introduce three important extensions to the voter model. First, we allow for the presence of the so-called zealots ([29–33], reviewed in [61]). Zealots are agents who never change their mind, and as such are permanent senders of a particular partisan position. They represent the presence of (and exposure to) partisan information sources that persistently advocate in favour or against vaccination (e.g. stubborn individuals, national health authorities, newspapers, Internet platforms, etc.). Second, we introduce a spatial structure. In the basic form of the voter model used here, interaction happens only within a geographically determined area (also called a patch). In our spatial model, exposure to other opinions is also possible between individuals who live in neighbouring patches (in addition to one’s own patch). Third, we allow for reinforcement of current opinions. Behavioural research documents that humans are more likely to engage with people who have similar behaviour or belief as themselves, and that they tend to discredit opinions that are not aligned with their own [62]. In our model, reinforcement enables the emergence of echo chambers.

We study the dynamics of opinion, i.e. the frequency of a certain opinion—pro-vaccine or anti-vaccine—per patch and across patches. As our model is stochastic by nature, we first analyse a deterministic limit which makes explicit the long-term behaviour of the model, that is whether one or both opinions can be maintained and what is the expected frequency of each opinion in each patch. Second, we use a weak-effects limit to obtain the expected distribution of opinion frequencies within and across patches. This so-called invariant measure will later be used for data analysis.

### 2.2. Model description

In order to represent the spatial structure of districts, we define Γ as an undirected graph. We write k∈Γ to indicate that *k* is a node of the graph, with each node representing a geographical unit, e.g. a patch or in our real data a district. For two nodes $k,k^{\prime }\in \Gamma$, we define the relation *k* ∼ *k*′ if and only if there is an edge between *k* and *k*′. Let *d*_*k*_ denote the degree of a given node k∈Γ, that is the number of neighbouring nodes. The nodes (patches/districts) themselves are assumed to have an identical social structure, and an identical population size. Let *N* denote the total population size in one district, *N*_*i*_ the number of zealots with opinion *i* ∈ {1, 2}, and *n*_*i*_ = *N*_*i*_/*N* the scaled number of *i*-zealots in a given district. Note that zealots rather represent a certain (pro-vaccine and anti-vaccine) information intensity, and do not represent real persons, that is, the population size *N* does not cover zealots, but only persons who can change their mind. Furthermore, let $X_{t}^{\left(k\right)}$ be the number of (non-stubborn) opinion 1 supporters (pro-vaccine) in node k∈Γ at time *t*, while $N-X_{t}^{\left(k\right)}$ is the number of (non-stubborn) opinion 2 (anti-vaccine) supporters.

Individuals change their opinion by being exposed to other opinions. A given individual changes their opinion with probability *μ*. In the zealot model without any spatial structure, the individual simply interacts with a randomly chosen other person (including the zealots) from their own district, and may adopt that person’s opinion (with probability *μ*). In our spatially structured, graph-based model, a person first decides if they interact with a neighbouring district (probability *τ*) or with a person of their own district (probability 1 − *τ*). In the case of interaction with a neighbouring district, each neighbouring district has the same chance to be selected. The resulting effective number of opinion 1 supporters our given individual is interacting with thus reads

$${\hat{X}}_{t}^{\left(k\right)}=(1-\tau )X_{t}^{\left(k\right)}+\tau {\overset{ˇ}{X}}^{\left(k\right)},\;{\overset{ˇ}{X}}^{\left(k\right)}\;:=\frac{1}{d_{k}}\sum_{k^{\prime }∼k}X_{t}^{\left(k^{\prime }\right)}.$$

The parameter *τ* represents the strength of communication and connectedness between neighbouring districts. A person thus interacts with an opinion 1 supporter (and thus adopts their opinion) with probability

$$\frac{{\hat{X}}_{t}^{\left(k\right)}+N_{1}}{N_{1}+N_{2}+N}.$$

We define the incremental increase or decrease, respectively, of the number of opinion 1 supporters as the spatial zealot model,

2.1 $$X_{t}^{\left(k\right)}\to X_{t}^{\left(k\right)}+1\;\text{at rate}\;\mu (N-X_{t}^{\left(k\right)})\;\frac{{\hat{X}}_{t}^{\left(k\right)}+N_{1}}{N_{1}+N_{2}+N}$$

and

2.2 $$X_{t}^{\left(k\right)}\to X_{t}^{\left(k\right)}-1\;\text{at rate}\;\mu X_{t}^{\left(k\right)}\;\frac{N-{\hat{X}}_{t}^{\left(k\right)}+N_{2}}{N_{1}+N_{2}+N}.$$

A person in an echo chamber is less likely to flip to the opposite opinion, be it because it is less likely to meet people of the opposite opinion, or because that person discards the alternative opinion as nonsense. We subsume both reasons as a decrease in the effectiveness of interaction: let $\vartheta_{1}$ denote the probability for an opinion 2 person to interact effectively with an opinion 1 supporter. ‘Effective interaction’ is an interaction in which the focal individual is open to adopting the opinion of their interaction partner. Let us explain this concept in more detail: when an anti-vaccine talks to a pro-vaccine, the anti-vaccine often does not listen to the pro-vaccine’s arguments at all. Therefore, in such a discussion the anti-vaccine cannot change their opinion, and in that sense the discussion is not ‘effective’. There are relatively few people among the pro-vaccine that a given anti-vaccine respects enough to even consider their arguments (and thus potentially be convinced to get vaccinated). The number of effective contacts with pro-vaccine is thus reduced to the fraction $\vartheta_{1}$. An opinion 2 supporter interacts effectively with all opinion 2 supporters $\left(1-{\hat{X}}_{t}^{\left(k\right)}+N_{2}\right)$, and with the fraction $\vartheta_{1}$ of opinion 1 supporters $\left(\vartheta_{1}({\hat{X}}_{t}^{\left(k\right)}+N_{1})\right)$. With $\vartheta_{1}$ capturing the strength of reinforcement of opinion 2 supporters, the probability of an opinion 2 supporter becoming an opinion 1 supporter reads

$$\frac{\vartheta_{1}({\hat{X}}_{t}^{\left(k\right)}+N_{1})}{\vartheta_{1}({\hat{X}}_{t}^{\left(k\right)}+N_{1})+(N-{\hat{X}}_{t}^{\left(k\right)}+N_{2})}.$$

Similarly, we introduce $\vartheta_{2}$ as the probability for an opinion 1 supporter to interact effectively with an opinion 2 person. With this idea, we can define the increase of the number of opinion 1 supporters as the reinforcement model,

2.3 $$\mu (N-X_{t}^{\left(k\right)})\;\frac{\vartheta_{1}({\hat{X}}_{t}^{\left(k\right)}+N_{1})}{\vartheta_{1}({\hat{X}}_{t}^{\left(k\right)}+N_{1})+(N-{\hat{X}}_{t}^{\left(k\right)}+N_{2})},$$

and similarly the decrease,

2.4 $$\mu X_{t}^{\left(k\right)}\;\frac{\vartheta_{2}(N-{\hat{X}}_{t}^{\left(k\right)}+N_{2})}{\left({\hat{X}}_{t}^{\left(k\right)}+N_{1})+\vartheta_{2}(N-{\hat{X}}_{t}^{\left(k\right)}+N_{2}\right)}.$$

Note that the bounded confidence model and similar approaches [40,41,63], which represent the quantitative opinion of a person as a real number in [0, 1], implement an idea which is similar in spirit (though more complex in the mathematical technique). Only persons who have a similar opinion (their ‘opinion number’ differs at most by some *ɛ* > 0) will effectively discuss and influence the opposite person's opinion. That mechanism resembles our model, where the probability of effective interaction decreases if two people do not have the same opinion. In the analytical part of the results, we scale the model both to a deterministic limit and to a weak-effects limit, in order to obtain bifurcations, phase transitions and the invariant measure.

### 2.3. Vaccination data

We apply the reinforcement model with spatial structure to data on vaccination coverage for measles in Germany for child cohorts born in 2008–2012, by district and birth year. For those birth years, a district’s measles vaccination quota was measured using a consistent method across Germany, and is publicly available through the Robert Koch Institute (RKI) ([64,65] and electronic supplementary material). Measles is a highly infectious childhood disease [66], with rare but serious complications, such as measles pneumonia. The standard public health recommendation in Germany is a first measles vaccination for children at an age of 11–14 months, and a second shot at 15–23 months [67]. We focus our analysis on the first vaccination shot because it best captures deliberate vaccination denial (rather than negligence/forgetfulness). Since public health recommendations are clear and salient, and access to the vaccine is convenient and free of charge, we can consider parents’ decision to not have their child vaccinated as a direct reflection of their opinions towards vaccination (pro- or anti-vaccine). In the electronic supplementary material, appendix, we present a similar analysis using data on meningococci vaccinations.

### 2.4. Model fitting and statistical analysis

In order to estimate the parameters of our (spatial) reinforcement model using the measles or meningococci vaccination data across all 413 districts in Germany, we use a statistical method based on likelihood computation. Data analysis is performed in two steps. First, we estimate the parameters of the decoupled model (assuming *τ* = 0) and compare its empirical fit with and without reinforcement. We compute the exact likelihood $L=P(x^{\left(k\right)}=y^{\left(k\right)})$, with *x*^(*k*)^ being the fraction of vaccinated individuals in district k∈Γ. Second, we estimate the parameters of the spatial model (*τ* ≥ 0). In that case, we observe the data *y*^(*k*)^ in grid node *k*, but obtaining the exact likelihood comes at prohibitively high computational costs. Following [68–70], we approximate the likelihood by the product of the marginal probabilities for single nodes, conditioned on the state of all other nodes. This conditioned likelihood (pseudo-likelihood) allows us to compute the normalizing constant *C* by a one-dimensional integration (*C* is a key parameter defined in the proposition and theorem in the Results section). Note that this integration has to be performed for each node, but it is still faster and more practical to compute |Γ| one-dimensional integrals rather than one |Γ|-dimensional integral. Parameter estimations based on pseudo-likelihood and exact likelihood typically yield comparable results [70]. The general pseudo-likelihood formula can be written as follows:

$$\hat{L}=\prod_{k\in \Gamma }P(x^{\left(k\right)}=y^{\left(k\right)}\;|\;x^{\left(k^{\prime }\right)}=y^{\left(k^{\prime }\right)},\;k^{\prime }\neq k).$$

Finally, in order to assess the sensitivity of the vaccination coverage *v* to changes in the model parameters *p*, i.e. zealot strength (*N*_1_, *N*_2_) and reinforcement strength ($\vartheta_{1}$, $\vartheta_{2}$), we compute the respective elasticities (for a similar approach, see [71]),

$$e_{v,p}=\frac{\;p}{v}\;\frac{\partial v}{\partial p}.$$

The elasticity *e*_*v*,*p*_ is dimensionless and approximates the percentage change in vaccination coverage *v* in response to a percentage change in the respective parameter *p*.

## 3. Results

### 3.1. Analytical results

#### 3.1.1. Deterministic limit

To understand the general behaviour of the model, we first scale the model to a deterministic continuum limit, using the assumption that the population size of the nodes (districts) is large. In this case, the stochastic property of the model becomes negligible and we obtain the deterministic limit, whose resulting equation can be readily interpreted.

Before analysing the more complex two-patch systems, let us briefly consider the simplest possible case, namely a single patch with symmetric parameters (same number of zealots and same strength of reinforcement for both opinions). We show that, if reinforcement becomes strong enough, the patch undergoes a so-called pitchfork bifurcation, meaning that three outcomes can occur: opinion 1 dominates, opinion 2 dominates and both opinions coexist at intermediate frequencies in the patch (electronic supplementary material, section A.4). More precisely, with low levels of reinforcement, we find only one locally asymptotically stable state, in which one of the two opinions vanishes. By contrast, when reinforcement is high, we find three branches of stationary states, of which two are locally asymptotically stable (the single opinion states), and the middle one (coexistence of both opinions) is unstable.

**Figure 1.**
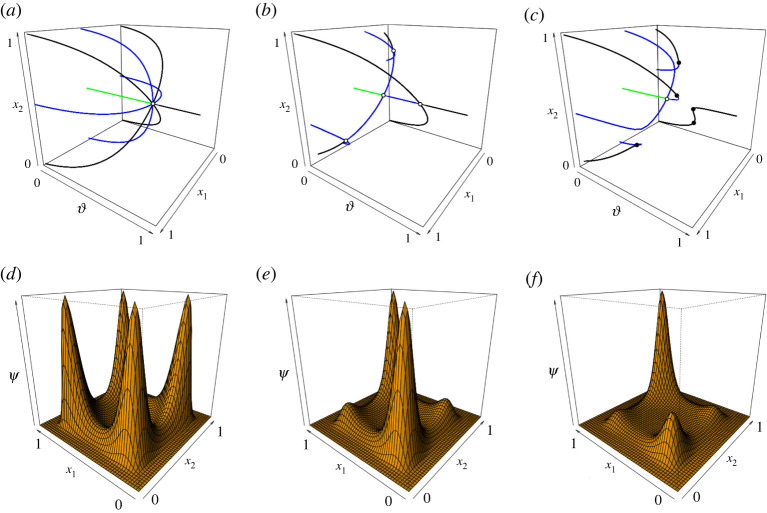
Upper row: Stationary states for the deterministic spatial two-patch model in dependency of the reinforcement parameter ϑ, with $\vartheta_{1}=\vartheta_{2}=\vartheta$. On the axes, *x*_*i*_ indicates the fraction of opinion 1 supporters in patch *i* ∈ {1, 2}. (*a*) Symmetric and decoupled *n*_1_ = *n*_2_ = 0.1, *τ* = 0. (*b*) Symmetric and connected, *n*_1_ = *n*_2_ = 0.1, *τ* = 0.1. (*c*) Non-symmetric and connected, *n*_1_ = 0.1, *n*_2_ = 0.105, *τ* = 0.1. Black: locally asymptotically stable states; blue/green: unstable states (one- and two-dimensional unstable manifold), closed circles: saddle-node bifurcations, open circles: (degenerated) pitchfork circles. Lower row: Invariant measure *ψ* for the stochastic spatial two-patch model, with *θ*_1_ = *θ*_2_ = 120. (*d*) Symmetric and decoupled *N*_1_ = *N*_2_ = 20, *γ* = *τ* = 0. (*e*) Symmetric and connected, *N*_1_ = *N*_2_ = 20, *γ* = 20. (*f*) Non-symmetric and connected, *N*_1_ = 20, *N*_2_ = 20.4, *γ* = 20.

These results are first extended to two patches without any interaction between them (*τ* = 0) and the outcome is illustrated in figure 1*a*–*c*, where *x*_1_ (*x*_2_) refers to the fraction of opinion 1 supporters in patch 1 (patch 2). The stationary states for the deterministic two-patch model is shown in dependency of the reinforcement parameter ϑ. Locally asymptotically stable (unstable) states are shown in black (blue: one-dimensional, green: two-dimensional unstable manifold), and bifurcation points are depicted as dots. If *τ* = 0, the two patches are decoupled (figure 1*a*). In this case, a product structure for the stationary states is found with each combination of single-patch stationary states yielding a valid stationary state for the two-patch situation. As three stationary states occur after the pitchfork bifurcation in a single patch, we find nine branches in the two-patch model after a highly degenerated pitchfork bifurcation. As both single-patch stationary states have to be locally stable to be combined into a locally stable two-patch state, we obtain four branches of stable stationary points: two of them are convergent (the same opinion prevails in both patches) and the other two are divergent (opposite opinions prevail in the two patches).

We can now extend the deterministic limit analysis to the two-patch model with interaction (*τ* > 0). The comparison between panels (*a*) and (*b*) of figure 1 illustrates that as interaction between patches increases (*τ* = 0.1), the degeneracy decreases (still under an identical set of parameter values for both opinions). The convergent stationary state *x*_1_ = *x*_2_ = 1/2 undergoes two subsequent non-degenerate pitchfork bifurcations. Particularly, the stable part of the divergent stationary solutions becomes smaller. If between-patch interaction increases even further, the divergent solutions eventually vanish as the interaction enforces a convergence of opinions. In other words, when a distinct communication happens between neighbouring districts, one of the two opinions prevails in both patches. If we break the symmetry of parameter values between opinions (figure 1*c*), the pitchfork bifurcations are unfolded into a series of saddle-node bifurcations. In the view of the multi-stable situation characterizing the deterministic behaviour, we expect—when accounting for stochasticity—the invariant measure to show a multi-modal distribution.

#### 3.1.2. Weak-effects limit

We now present results for the stochastic model under the weak-effects limit. We aim for the analysis of patch data, where each patch has a certain population size. Experience, e.g. in population genetics [72,73], indicates that this limit is better suited for the analysis of such data than the deterministic limit. For the weak-effects limit, we choose a different scaling of the parameters than before. We assume that the number of zealots *N*_*i*_ are independent of the population size *N*, and that the reinforcement component becomes small for large *N*, $\vartheta_{i}=1-\theta_{i}/N$. Note that in the original scaling, a small value of $\vartheta_{i}$ indicates a strong reinforcement, while from now on (with the new scaling), large values of *θ*_*i*_ indicate strong reinforcement. We furthermore allow the spatial interaction parameter *τ* to depend on 1/*N* but also on the frequency of a certain opinion in a given patch, with the new parameter *γ* as proportionality constant

$$\tau (x^{\left(k\right)})=\frac{1}{N}\;\;\gamma \;\;x^{\left(k\right)}\;\;(1-x^{\left(k\right)}).$$

Interaction *per se* is assumed to be a weak effect, so that *τ*(*x*^(*k*)^) tends to zero if *N* tends to infinity. Moreover, if almost all individuals in a given patch have the same opinion (*x*^(*k*)^ ≈ 1 or *x*^(*k*)^ ≈ 0), interaction has almost no effect. In other words, the effect of interaction between patches is maximized if a patch is maximally heterogeneous, i.e. if *x*^(*k*)^ ≈ 1/2. Our assumptions reflect the application of the model. Put simply, we assume that if all my neighbours have the same opinion as myself, I am inclined to follow that opinion too, and do not look further to cross-check my opinion with people from other districts (interaction parameter *τ* is very small). By contrast, if my neighbours give me contradicting pieces of advice, I may be more inclined to ask additional people. Consequently, I am more likely to also communicate with individuals from neighbouring patches (*τ* is larger). Moreover, our choice of *τ*(.) is mathematically convenient as it yields a consistent scaling and an appropriate invariant measure.

Before we investigate the spatial model (with interaction between neighbouring districts), we first derive the invariant distribution for the decoupled model (without interaction between neighbouring patches). While the proof of the next proposition can be found in [74], we sketch the proof in the electronic supplementary material, appendix for completeness.

**Proposition 3.1.**

*Let* *N*_*i*_ *denote the number of zealots for group* *i*, *N* *the population size, and* $\vartheta_{i}=1-\theta_{i}/N$ *the parameter describing reinforcement. We assume no interaction*, *γ* = 0, *such that all patches become independent and identical. In the limit* *N* → ∞, *the density of the invariant measure for the random variable* $x_{t}=X_{t}^{\left(k\right)}/N$ (*for any* *k*) *is given by*

3.1 $$\varphi (x)=C\;e^{(1/2)(\theta_{1}+\theta_{2})x^{2}-\theta_{1}\;x}\;\;x^{N_{1}-1}\;{\left(1-x\right)}^{N_{2}-1},$$

*where* *C* *is determined by the condition that the integral over* φ(.) *is* 1.

Using this result, we can determine the invariant distribution for the spatial model, yielding our main theorem below. We are particularly interested in the invariant measure *ψ*(.) which summarizes the pseudo-equilibrium distribution of the frequencies of opinions in a stochastic model.

**Theorem 3.2.**

*Let* *N*_*i*_ *denote the number of zealots for group* *i*, *N* *the population size, and* $\vartheta_{i}=1-\theta_{i}/N$ *the parameter describing reinforcement. We also scale the spacial interaction strength* $\tau =1/N\;\;\gamma \;\;x^{\left(k\right)}\;\;(1-x^{\left(k\right)})$. *In the limit* *N* → ∞, *the density of the invariant measure for the random variable* $x_{t}^{\left(.\right)}=(x_{t}^{\left(1\right)},\ldots ,$ $x^{\left(|\Gamma |\right)})=(X_{t}^{\left(1\right)}/N,\ldots ,X_{t}^{\left(|\Gamma |\right)}/N)$ *is given by*

3.2 $$\psi (x^{\left(\cdot \right)})=C\;\prod_{k\in \Gamma }\left(\varphi (x^{\left(k\right)})\exp\;\left\{-\frac{\gamma }{4\;d_{k}}\sum_{k^{\prime }∼k}{\left(x^{\left(k\right)}-x^{\left(k^{\prime }\right)}\right)}^{2}\right\}\right),$$

*where* φ(.) *is the homogeneous-population distribution defined in equation* (*3.1*), *and* *C* *is determined by the condition that the integral over* *ψ*(.) *is* 1.

The distribution of opinion frequencies has a multiplicative structure, where the first term is related to the local dynamics within a given patch *k* (φ(*x*^(*k*)^)), while the second term accounts for the communication between neighbouring patches. For the two-patch system, the shape of the invariant distribution can be visualized (figure 1). The local maxima are the stochastic analogue of the locally asymptotically stable stationary points in the deterministic case. In fact, figure 1*d*–*f* corresponds to figure 1*a*–*c* for appropriate choices of ϑ. We represent in subfigure (*d*) the case without patch interaction for which four local maxima appear, mimicking the deterministic results of four stable stationary points. If interaction strength increases (*γ* > 0 defining *τ*), the non-symmetric maxima decrease, while the symmetric maxima (on the line *x*_1_ = *x*_2_) are still present (subfigure (*e*)). If we choose non-symmetric parameters ($N_{1}\neq N_{2}$ in subfigure (*f*)), we still find the local maxima on the diagonal, but now one maximum dominates the distribution. To conclude, the different parameters (strength of reinforcement, strength of zealots, interaction between patches) strongly influence the shape of opinion frequency distributions. All parameters being identifiable, we set to estimate the parameters of our model based on real-world data.

### 3.2. Vaccination data analysis

**Figure 2.**
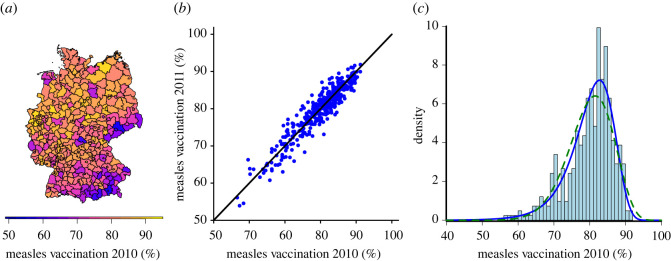
(*a*) Measles vaccination coverage (in per cent) for children born in 2010 across 413 districts in Germany (GIS-data: © GeoBasis-DE/BKG (2021)). (*b*) Vaccination coverage for birth years 2010 and 2011, by district; 45° line for comparison. (*c*) Histogram of vaccination coverage for measles per district in 2010. The solid blue (dashed green) line: fit of the decoupled model with (without) reinforcement. Bin size is 0.01.

The RKI defines a district’s vaccination quota as the number of children born in year X getting their first measles shot within their first 24 months, divided over all children born in year X. The left panel of figure 2 illustrates the variation of vaccination quotas across Germany. Visually, the data suggest the existence of spatial correlations, e.g. a large cluster with a low vaccination coverage in southern Germany. Moreover, as shown in the middle panel of figure 2, districts’ vaccination quotas are strikingly consistent across birth years. In our dataset, the smallest year-to-year correlation is *ρ* = 0.87 (Spearman correlation coefficient). This resonates with our assumption that people’s local environment is central for their opinion formation and thus for their decision to support or reject vaccination.

We assess the extent to which reinforcement explains the distribution of vaccine opinions across districts in Germany by estimating the reinforcement parameter *θ*_*i*_. Figure 2*c* illustrates the superior fit of the decoupled model with reinforcement rather than without reinforcement. The model without reinforcement is rejected (Kolmogorow–Smirnow test, significance levels *p* < 0.05 for all birth years separately). By contrast, the model with reinforcement is appropriate for the data (Kolmogorow–Smirnow test, significance levels *p* > 0.05 for all birth years separately), and the reinforcement component is indispensable for explaining the data (the likelihood-ratio test rejects the model without reinforcement, *p* < 0.000005, see electronic supplementary material, appendix for details).

**Figure 3.**
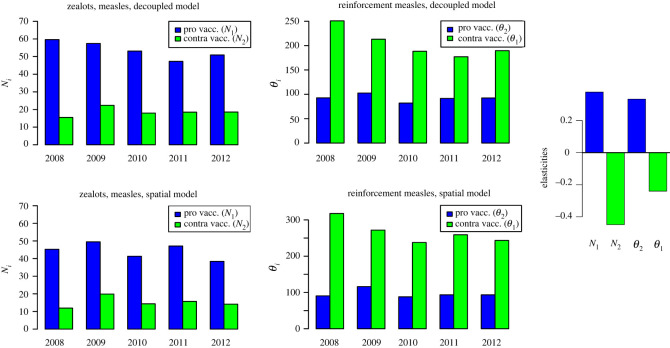
Estimation parameters for the decoupled model (upper panels) and the spatial model (lower panels). Right panel: Elasticities for the decoupled model (parameters of measles 2010). Blue: pro-vaccination parameters, green: anti-vaccination parameters.

The upper panels of figure 3 display the estimated model parameters for the decoupled model, for each birth year separately. We find the zealot parameter to be larger for the pro-vaccination than for the anti-vaccination opinion. By contrast, the reinforcement parameter is larger for the anti-vaccination than for the pro-vaccination opinion. This pattern is consistent across all birth years. Additionally allowing for interaction between neighbouring districts is meaningful (the estimated *γ* is consistently positive) but does not affect the general patterns found above. In fact, the upper panels (decoupled model) and the lower panels of figure 3 (spatial model) are strikingly similar. The zealot component is consistently larger for the pro-vaccination opinion whereas the reinforcement component is larger for the anti-vaccination opinion. This suggests that individuals are more often exposed to information that promotes vaccination, but vaccination deniers are more likely to filter out this pro-information and focus on anti-information. In the electronic supplementary material, appendix, we show that the relative sizes of all four parameters are robust to applying the model to meningococci vaccinations rather than measles.

As shown in figure 3, right panel, we find, as intuitively expected, that increasing any of the two pro-vaccination parameters (*N*_1_, *θ*_2_) leads to an increase of the vaccination coverage, while increasing the anti-vaccination parameters (*N*_2_, *θ*_1_) entails a decrease. More interestingly, we find that decreasing the zealot strength of vaccination-deniers *N*_2_ has the strongest effect on vaccination uptake. By contrast, influencing the reinforcement parameter of vaccination deniers has the smallest impact.

## 4. Discussion

We have developed and analysed a spatial opinion model that incorporates social interactions by reinforcement, thus allowing for the occurrence of echo chambers. Applying our model to fit and draw parameter inference from vaccination data for measles and meningococci, we find our reinforcement model to be highly superior to a model without reinforcement. Estimated parameters are robust across both diseases and all birth years in our sample, and to allowing for spatial correlations between neighbouring districts.

The model introduced here is capable of structuring the factors influencing vaccination hesitancy into (i) exposure to—potentially distorted—partisan information and (ii) people’s tendency to consume information that is aligned with their worldview; and to allow these components to be measured with spatial data. We find the zealot component to be much larger for the pro-vaccine opinion than for the anti-vaccine opinion. This resonates with the idea that in Germany the pro-vaccine opinion has much more exposure than the anti-vaccine opinion. By contrast, the reinforcement component is much larger for the anti-vaccine opinion. This is consistent with empirical studies that associate vaccine hesitancy with people’s receptiveness to populism and conspiracy theories [74–76]. We find that the model describes well the aggregated vaccination data. Even though our model is based on well-documented socio-psychological mechanisms on the micro-level (behaviour of individuals), it is of course possible that the aggregated data studied is insensitive to micro-level mechanisms. In that case, the model would only be valid at the population level.

By computing elasticities, our model can be used to predict the effectiveness of different measures aiming at increasing vaccination uptake. We find that the most sensitive parameter influencing vaccination uptake is exposure to the anti-vaccine opinion. According to our model, measures aiming at reducing the salience of partisan anti-vaccine information sources would have the largest effect on enhancing vaccination uptake. By contrast, we find that measures aiming at reducing the reinforcement of vaccination deniers have the smallest impact. This resonates with the idea that dialogue-based approaches which are carefully targeted to a specific social group are among the most effective measures against vaccination hesitancy [5]. Studying vaccine hesitancy for COVID-19, Klüver *et al.* [77] find positive information, appropriate communication, and trust to be central. If the behavioural mechanisms behind people’s reluctance to receive a COVID-19 vaccination are similar to their opposition to vaccines against measles and meningococci, our study suggests that targeting the partisan spreaders of anti-vaccine information could be very effective. An important difference between COVID-19 vaccinations on the one hand, and measles and meningococci vaccinations on the other hand, is the incidence of the disease. Both measles and meningitis have been extremely rare in Germany for many years. Consequently, our model neglects the influence of the disease incidence on the vaccination rate, as well as the effect of the vaccination coverage on the infection dynamics [22]. In future research, our model could be generalized to investigate vaccination opinion dynamics for prevalent diseases.

Trust in government institutions is instrumental for how people process government recommendations, and thus for their willingness to get vaccinated (and to vaccinate their children). By capturing well-documented behavioural biases in people’s processing of information, and delivering testable predictions at the macro level, our model complements the growing empirical literature on the historical determinants of impersonal trust [78]. Our model explains the substantial differences in measles and meningococci vaccination coverage within Germany—both within the former West Germany and within the former East Germany—by the occurrence of the anti-vaccination opinion driven by strong echo chambers. Further historical and sociological factors might contribute to the spatial correlations in vaccination coverage. The opinion dynamics and echo chamber mechanisms we model here do not contradict the existence of historical factors. On the contrary, the propensity of people to influence opinion dynamics (being a zealot) or to be influenced by opinion dynamics (adhering to an echo chamber) may probably have deeper roots into local historical factors. For Germany in particular, regions with high intensity of East German state surveillance in the 1980s have been shown to have lower current levels of impersonal trust and lower election turnout [79], and municipalities with high support for the Nazi party in 1933 are more likely to vote for the populist AfD today [80].

Beyond vaccination hesitancy, our model is applicable to a large range of problems of opinion polarization; from discrepancies about the extent and causes of global warming, to perceptions of inequality and the role of government [81–83]. In psychology, economics and political science, a prominent explanation for polarization has been the idea of motivated reasoning [54,84,85]: when people are emotionally invested in a certain state of the world being true—e.g. because it favours them economically, or because their partisan alignment is a critical part of their identity [62]—it limits their ability to process information in an unbiased manner. While our model considers the behavioural biases of the *receivers* of information, it neglects the motives of the *senders* of information [86,87]. In a world that relies increasingly on highly specialized expert knowledge, while at the same time citizens’ trust in experts is being constantly undermined by populist leaders and media outlets, future work should expand our model to capture the interplay between expert senders and non-expert receivers of information.

Finally, we comment on a more technical aspect of our model. We introduced the model as a branching process at the level of single individuals. Looking at a deterministic limit, we found a sequence of pitchfork bifurcations. Given appropriate parameters, these bifurcations collapsed into a highly degenerate pitchfork bifurcation at a single point. This bifurcation structure resembles that of the Ising/Curie–Weiss model, which has also been applied to opinion dynamics [36]. Yet, the central mechanism of our model is fundamentally different. While the Ising/Curie–Weiss model implements a majority rule [88], our reinforcement model with spatial structure is—just as the basic voter model—based on pairwise interactions. We show that a different scaling of the parameters yields the weak-effects limit. This limit is a stochastic process that is independent of the population size. It is thus suited to analyse data of populations of an appropriate size. Nevertheless, if the population within a patch is too small, the branching process is better suited but stochastic noise can be very strong, decreasing the power of parameter inference. If the population is too large, the noise averages out, and we approach a deterministic model. Future work should investigate for which geographical/administrative units our model is best suited in order to gauge its applicability to different countries and different opinion frequencies.

## Data Availability

The data are freely available from the pages of the Robert Koch Institute, Germany. A full reference is given in the electronic supplementary material [89]. In detail: the web pages for the measles data are located in the URLs https://www.versorgungsatlas.de/themen/versorgungsprozesse?tab=3&uid=76&cHash=15379e83482f9325cf011f690c059c26 (accessed 2 May 2021), https://www.versorgungsatlas.de/themen/versorgungsprozesse?tab=3&uid=43&cHash=31605ab96524f1a101ec8e3aac07e388 (accessed 2 May 2021) and that for meningoccoci https://www.versorgungsatlas.de/themen/versorgungsprozesse?tab=4&uid=75&cHash=08f43064449be9c3947a6ffd61e0e09a (accessed 2 May 2021) The GIS data used are available at the URL https://gdzshopv-lpz.bkg.bund.de/index.php/default/catalog/product/view/id/773/s/nuts-gebiete-1-250-000-stand-01-01-nuts250-01-01/category/8/?_store=default.

## References

[1] Bozzola E, Spina G, Tozzi AE, Villani A. 2020 Global measles epidemic risk: current perspectives on the growing need for implementing digital communication strategies. Risk Manage. Healthcare Policy **13**, 2819-2826. (10.2147/RMHP.S201279) PMC7725071 33312006

[2] Dubé E, Laberge C, Guay M, Bramadat P, Roy R, Bettinger JA. 2013 Vaccine hesitancy. Hum. Vacc. Immunotherapeut. **9**, 1763-1773. (10.4161/hv.24657) PMC3906279 23584253

[3] Siciliani L, Wild C, McKee M, Kringos D, Barry MM, Barros PP, Maeseneer JD, Murauskiene L, Ricciardi W. 2020 Strengthening vaccination programmes and health systems in the European Union: a framework for action. Health Policy **124**, 511-518. (10.1016/j.healthpol.2020.02.015) 32276852

[4] Wilder-Smith AB, Qureshi K. 2019 Resurgence of measles in Europe: a systematic review on parental attitudes and beliefs of measles vaccine. J. Epidemiol. Global Health **10**, 46. (10.2991/jegh.k.191117.001) PMC7310814 32175710

[5] Jarrett C, Wilson R, O’Leary M, Eckersberger E, Larson HJ. 2015 Strategies for addressing vaccine hesitancy – a systematic review. Vaccine **33**, 4180-4190. (10.1016/j.vaccine.2015.04.040) 25896377

[6] MacDonald NE. 2015 Vaccine hesitancy: definition, scope and determinants. Vaccine **33**, 4161-4164. (10.1016/j.vaccine.2015.04.036) 25896383

[7] Quinn SC, Jamison AM, Freimuth VS. 2019 Measles outbreaks and public attitudes towards vaccine exemptions: some cautions and strategies for addressing vaccine hesitancy. Hum. Vacc. Immunotherapeut. **16**, 1050-1054. (10.1080/21645515.2019.1646578) PMC7227729 31403354

[8] Shen S, Dubey V. 2019 Addressing vaccine hesitancy: clinical guidance for primary care physicians working with parents. Can. Fam. Physician **65**, 175-181. 30867173 PMC6515949

[9] Storr C, Sanftenberg L, Schelling J, Heininger U, Schneider A. 2018 Measles status—barriers to vaccination and strategies for overcoming them. *Deutsches Aerzteblatt Online.* See https://www.aerzteblatt.de/int/archive/article/202016. 10.3238/arztebl.2018.0723 PMC6293121 30518471

[10] Funk S, Salathé M, Jansen VAA. 2010 Modelling the influence of human behaviour on the spread of infectious diseases: a review. J. R. Soc. Interface **7**, 1247-1256. (10.1098/rsif.2010.0142) 20504800 PMC2894894

[11] Yaqub O, Castle-Clarke S, Sevdalis N, Chataway J. 2014 Attitudes to vaccination: a critical review. Soc. Sci. Med. **112**, 1-11. (10.1016/j.socscimed.2014.04.018) 24788111

[12] Fine PEM, Clarkson JA. 1986 Individual versus public priorities in the determination of optimal vaccination policies. Am. J. Epidemiol. **124**, 1012-1020. (10.1093/oxfordjournals.aje.a114471) 3096132

[13] McDonald SA, van Lier A, Plass D, Kretzschmar ME. 2012 The impact of demographic change on the estimated future burden of infectious diseases: examples from hepatitis B and seasonal influenza in the Netherlands. BMC Public Health **12,** 1046. (10.1186/1471-2458-12-1046) PMC3537516 23217094

[14] Bauch CT, Galvani AP, Earn DJD. 2003 Group interest versus self-interest in smallpox vaccination policy. Proc. Natl Acad. Sci. USA **100**, 10 564-10 567. (10.1073/pnas.1731324100) 12920181 PMC193525

[15] Fu F, Rosenbloom DI, Wang L, Nowak MA. 2010 Imitation dynamics of vaccination behaviour on social networks. Proc. R. Soc. B **278**, 42-49. (10.1098/rspb.2010.1107) PMC2992723 20667876

[16] Galvani AP, Reluga TC, Chapman GB. 2007 Long-standing influenza vaccination policy is in accord with individual self-interest but not with the utilitarian optimum. Proc. Natl Acad. Sci. USA **104**, 5692-5697. (10.1073/pnas.0606774104) 17369367 PMC1838447

[17] Müller J. 1997 Optimal vaccination strategies–for whom? Math. Biosci. **139**, 133-154. 9009575 10.1016/s0025-5564(96)00140-x

[18] Alvarez-Zuzek LG, Rocca CEL, Iglesias JR, Braunstein LA. 2017 Epidemic spreading in multiplex networks influenced by opinion exchanges on vaccination. PLoS ONE **12**, e0186492. (10.1371/journal.pone.0186492) 29121056 PMC5679524

[19] Pires MA, Oestereich AL, Crokidakis N. 2018 Sudden transitions in coupled opinion and epidemic dynamics with vaccination. J. Stat. Mech.: Theory Exp. **2018**, 053407. (10.1088/1742-5468/aabfc6)

[20] Salathé M, Bonhoeffer S. 2008 The effect of opinion clustering on disease outbreaks. J. R. Soc. Interface **5**, 1505-1508. (10.1098/rsif.2008.0271) 18713723 PMC2607358

[21] Basu S, Chapman GB, Galvani AP. 2008 Integrating epidemiology, psychology, and economics to achieve HPV vaccination targets. Proc. Natl Acad. Sci. USA **105**, 19 018-19 023. (10.1073/pnas.0808114105) PMC2596236 19015536

[22] Bauch CT, Bhattacharyya S. 2012 Evolutionary game theory and social learning can determine how vaccine scares unfold. PLoS Computat. Biol. **8**, e1002452. (10.1371/journal.pcbi.1002452) PMC3320575 22496631

[23] Rao N, Möbius MM, Rosenblat T. 2007 Social networks and vaccination decisions. Working Papers 07–12. Boston, MA: Federal Reserve Bank of Boston.

[24] Bhattacharyya S, Vutha A, Bauch CT. 2019 The impact of rare but severe vaccine adverse events on behaviour-disease dynamics: a network model. Sci. Rep. **9**, 1-13. (10.1038/s41598-019-43596-7) 31073195 PMC6509123

[25] Eames KTD. 2009 Networks of influence and infection: parental choices and childhood disease. J. R. Soc. Interface **6**, 811-814. (10.1098/rsif.2009.0085) 19447820 PMC2820361

[26] Velásquez-Rojas F, Vazquez F. 2017 Interacting opinion and disease dynamics in multiplex networks: discontinuous phase transition and nonmonotonic consensus times. Phys. Rev. E **95**, 052315. (10.1103/physreve.95.052315) PMC7219934 28618582

[27] Carpentras D, Lüders A, Quayle M. 2022 Mapping the global opinion space to explain anti-vaccine attraction. Sci. Rep. **12**, 1-9. (10.1038/s41598-022-10069-3) 35589806 PMC9120185

[28] Liggett T. 1985 Interacting particle systems. Berlin, Germany: Springer.

[29] Braha D, de Aguiar MA. 2017 Voting contagion: modeling and analysis of a century of US presidential elections. PLoS ONE **12**, e0177970. (10.1371/journal.pone.0177970) 28542409 PMC5436881

[30] Fernández-Gracia J, Suchecki K, Ramasco JJ, San Miguel M, Eguíluz VM. 2014 Is the voter model a model for voters? Phys. Rev. Lett. **112**, 158701. (10.1103/physrevlett.113.089903) 24785078

[31] Galam S. 2015 Stubbornness as an unfortunate key to win a public debate: an illustration from sociophysics. Mind Soc. **15**, 117-130. (10.1007/s11299-015-0175-y)

[32] Galam S, Jacobs F. 2007 The role of inflexible minorities in the breaking of democratic opinion dynamics. Physica A **381**, 366-376. (10.1016/j.physa.2007.03.034)

[33] Mobilia M. 2013 Commitment versus persuasion in the three-party constrained voter model. J. Stat. Phys. **151**, 69-91. (10.1007/s10955-012-0656-x)

[34] Sznajd-Weron K. 2005 Sznajd model and its applications. Acta Phys. Pol. B **36**, 2537.

[35] Sznajd-Weron K, Sznajd J. 2000 Opinion evolution in closed community. Int. J. Mod. Phys. C **11**, 1157-1165. (10.1142/S0129183100000936)

[36] Nicolao L, Ostilli M. 2019 Critical states in political trends. How much reliable is a poll on twitter? The Potts model and the inverse problem in Social Science. Physica A **533**, 121920. (10.1016/j.physa.2019.121920)

[37] Axelrod R. 1997 The dissemination of culture: a model with local convergence and global polarization. J. Conflict Resolut. **41**, 203-226. (10.1177/0022002797041002001)

[38] Waagen A, Verma G, Chan K, Swami A, D’Souza R. 2015 Effect of zealotry in high-dimensional opinion dynamics models. Phys. Rev. E **91**, 022811. (10.1103/PhysRevE.91.022811) 25768556

[39] Xie J, Emenheiser J, Kirby M, Sreenivasan S, Szymanski BK, Korniss G. 2012 Evolution of opinions on social networks in the presence of competing committed groups. PLoS ONE **7**, e33215. (10.1371/journal.pone.0033215) 22448238 PMC3308977

[40] Dong Y, Zhan M, Kou G, Ding Z, Liang H. 2018 A survey on the fusion process in opinion dynamics. Inf. Fusion **43**, 57-65. (10.1016/j.inffus.2017.11.009)

[41] Hegselmann R, Krause U. 2002 Opinion dynamics and bounded confidence models, analysis, and simulation. J. Artif. Soc. Soc. Simul. **5**, 3/2.

[42] Duggins P. 2017 A psychologically-motivated model of opinion change with applications to American politics. JASSS **20**, 1/13. (10.18564/jasss.3316)

[43] Engel C, Kube S, Kurschilgen M. 2021 Managing expectations: how selective information affects cooperation and punishment in social dilemma games. J. Econ. Behav. Organ. **187**, 111-136. (10.1016/j.jebo.2021.04.029)

[44] Simonov A, Sacher SK, Dubé J-PH, Biswas S. 2020 The persuasive effect of Fox News: non-compliance with social distancing during the COVID-19 pandemic. Technical report. Cambridge, MA: National Bureau of Economic Research.

[45] Enke B. 2020 What you see is all there is. Q. J. Econ. **135**, 1363-1398. (10.1093/qje/qjaa012)

[46] Pariser E. 2011 The filter bubble: what the Internet is hiding from you. New York, NY: Penguin Press.

[47] Sunstein C. 2011 Echo chambers: Bush v. Gore, impeachment, and beyond. Princeton, NJ: Princeton University Press.

[48] Borgesius FJZ, Trilling D, Möller J, Bodó B, de Vreese CH, Helberger N. 2016 Should we worry about filter bubbles? Internet Policy Rev. **5**, 1-16. (10.14763/2016.1.401)

[49] Flaxman S, Goel S, Rao JM. 2016 Filter bubbles, echo chambers, and online news consumption. Public Opin. Q. **80**, 298-320. (10.1093/poq/nfw006)

[50] Bartlett F. 1932 Remembering: a study in experimental and social psychology. Cambridge, UK: Cambridge University Press.

[51] Festinger L. 1957 A theory of cognitive dissonance. Stanford, CA: Stanford University Press.

[52] Taylor SE, Crocker J. 1981 Schematic bases of social information processing. In *Social cognition: The Ontario Symposium* (eds ET Higgins, P Hermann, MP Zanna), pp. 89–134. Hillsdale, NJ: Lawrence Erlbaum Associates.

[53] Fryer Jr RG, Harms P, Jackson MO. 2019 Updating beliefs when evidence is open to interpretation: implications for bias and polarization. J. Eur. Econ. Assoc. **17**, 1470-1501. (10.1093/jeea/jvy025)

[54] Kahan DM. 2012 Ideology, motivated reasoning, and cognitive reflection: an experimental study. Judgm. Decis. Mak. **8**, 407-424.

[55] Cohen GL. 2003 Party over policy: the dominating impact of group influence on political beliefs. J. Pers. Soc. Psychol. **85**, 808-822. (10.1037/0022-3514.85.5.808) 14599246

[56] Tajfel H, Turner J. 1986 The social identity theory of intergroup behaviour. In *Psychology of intergroup relations* (eds S Worchel, WG Austin), The Nelson-Hall Series in Psychology, pp. 7–24. Chicago, IL: Nelson-Hall.

[57] Krupenkin M. 2021 Does partisanship affect compliance with government recommendations? Polit. Behav. **43**, 451-472. (10.1007/s11109-020-09613-6) 32421091 PMC7224154

[58] Allcott H, Boxell L, Conway J, Gentzkow M, Thaler M, Yang D. 2020 Polarization and public health: partisan differences in social distancing during the Coronavirus pandemic. J. Public Econ. **191**, 104254. (10.1016/j.jpubeco.2020.104254) 32836504 PMC7409721

[59] Goffrier B, Schulz M, Bätzing-Feigenbaum J. 2017 Analyse des räumlichen Zusammenhangs zwischen den Impfquoten der Masern- und Meningokokken-C-Impfungen. *Zentralinstitut für die kassenärztliche Versorgung in Deutschland (Zi), Versorgungsatlas-Bericht Nr. 17/07*. (10.20364/VA-17.07)

[60] Cinelli M, De Francisci Morales G, Galeazzi A, Quattrociocchi W, Starnini M. 2021 The echo chamber effect on social media. Proc. Natl Acad. Sci. USA **118**, e2023301118. (10.1073/pnas.2023301118) 33622786 PMC7936330

[61] Castellano C, Fortunato S, Loreto V. 2009 Statistical physics of social dynamics. Rev. Mod. Phys. **81**, 591-646. (10.1103/RevModPhys.81.591)

[62] Iyengar S, Lelkes Y, Levendusky M, Malhotra N, Westwood SJ. 2019 The origins and consequences of affective polarization in the United States. Annu. Rev. Polit. Sci. **22**, 129-146. (10.1146/annurev-polisci-051117-073034)

[63] Deffuant G, Neau D, Amblard F, Weisbuch G. 2001 Mixing beliefs among interacting agents. Adv. Complex Syst. **3**, 87-98. (10.1142/S0219525900000078)

[64] Goffrier B, Schulz M, Bätzing-Feigenbaum J. 2016 Masernimpfungen gemäß STIKO-Empfehlungen anhand vertragsärztlicher Abrechnungsdaten von 2009 bis 2014. (10.20364/VA-16.07)

[65] Schulz M, Mangiapane S. 2013 Masernimpfungen bei Kindern bis zu einem Alter von zwei Jahren. See https://www.versorgungsatlas.de/fileadmin/ziva_docs/43/Bericht_Masernimpfung.pdf.

[66] Guerra FM, Bolotin S, Lim G, Heffernan J, Deeks SL, Li Y, Crowcroft NS. 2017 The basic reproduction number (r0) of measles: a systematic review. Lancet Infect. Dis. **17**, e420-e428. (10.1016/S1473-3099(17)30307-9) 28757186

[67] Robert Koch Institut. 2021 RKI Ratgeber Masern. See https://www.rki.de/DE/Content/Infekt/EpidBull/Merkblaetter/Ratgeber_Masern.html.

[68] Anderson CJ, Wasserman S, Crouch B. 1999 A p* primer: logit models for social networks. Soc. Netw. **21**, 37-66. (10.1016/S0378-8733(98)00012-4)

[69] Frank O, Strauss D. 1986 Markov graphs. J. Am. Stat. Soc. **81**, 832-842. (10.1080/01621459.1986.10478342)

[70] Strauss D, Ikeda M. 1990 Pseudolikelihood estimation for social networks. J. Am. Stat. Assoc. **85**, 204-212. (10.1080/01621459.1990.10475327)

[71] Zi Z. 2011 Sensitivity analysis approaches applied to systems biology models. IET Syst. Biol. **5**, 336-346. (10.1049/iet-syb.2011.0015) 22129029

[72] Etheridge A. 2011 *Some mathematical models from population genetics*. LNM 2012. Berlin, Germany: Springer.

[73] Tavaré S. 2004 Ancestral inference in population genetics. In *Lectures on probability theory and statistics* (ed. J Picard), pp. 3–180. LNM 1837. Berlin, Germany: Springer.

[74] Müller J, Hösel V, Tellier A. 2020 Filter bubbles, echo chambers, and reinforcement: tracing populism in election data. (http://arxiv.org/abs/2007.03910)

[75] Kennedy J. 2019 Populist politics and vaccine hesitancy in western Europe: an analysis of national-level data. Eur. J. Public Health **29**, 512-516. (10.1093/eurpub/ckz004) 30801109

[76] Stecula DA, Pickup M. 2021 How populism and conservative media fuel conspiracy beliefs about COVID-19 and what it means for COVID-19 behaviors. Res. Polit. **8**, 205316802199397. (10.1177/2053168021993979)

[77] Klüver H, Hartmann F, Humphreys M, Geissler F, Giesecke J. 2021 Incentives can spur COVID-19 vaccination uptake. Proc. Natl Acad. Sci. USA **118**, e2109543118. (10.1073/pnas.2109543118) 34413212 PMC8433545

[78] Alesina A, Giuliano P. 2015 Culture and institutions. J. Econ. Literat. **53**, 898-944. (10.1257/jel.53.4.898)

[79] Lichter A, Löffler M, Siegloch S. 2021 The long-term costs of government surveillance: insights from Stasi spying in east Germany. J. Eur. Econ. Assoc. **19**, 741-789. (10.1093/jeea/jvaa009)

[80] Cantoni D, Hagemeister F, Westcott M. 2019 Persistence and activation of right-wing political ideology. *Rationality & Competition Discussion Paper Series No. 143.* CRC TRR 190 Rationality and Competition.

[81] Alesina A, Miano A, Stantcheva S. 2020 The polarization of reality. AEA Papers and Proc. **110**, 324-328. (10.1257/pandp.20201072)

[82] Egan PJ, Mullin M. 2017 Climate change: US public opinion. Annu. Rev. Polit. Sci. **20**, 209-227. (10.1146/annurev-polisci-051215-022857)

[83] Kurschilgen M. 2021 Moral awareness polarizes people’s fairness judgments. *Munich Papers in Political Economy 2021/08*. Munich, Germany: TUM School of Governance at the Technical University of Munich.

[84] Bénabou R, Tirole J. 2016 Mindful economics: the production, consumption, and value of beliefs. J. Econ. Perspect. **30**, 141-164. (10.1257/jep.30.3.141)

[85] Zimmermann F. 2020 The dynamics of motivated beliefs. Am. Econ. Rev. **110**, 337-361. (10.1257/aer.20180728)

[86] Gneezy U, Saccardo S, Serra-Garcia M, van Veldhuizen R. 2020 Bribing the self. Games Econ. Behav. **120**, 311-324. (10.1016/j.geb.2019.12.010)

[87] Kurschilgen M, Marcin I. 2019 Communication is more than information sharing: the role of status-relevant knowledge. Games Econ. Behav. **113**, 651-672. (10.1016/j.geb.2018.11.007)

[88] Krapivsky PL, Redner S, Ben-Naim E. 2017 A kinetic view of statistical physics. Cambridge, UK: Cambridge University Press.

[89] Müller J, Tellier A, Kurschilgen M. 2022 Echo chambers and opinion dynamics explain the occurrence of vaccination hesitancy. *Figshare*. (10.6084/m9.figshare.c.6238123) PMC9554521 36312563

